# Concurrent miR-21 suppression and FXR activation as a mechanism of improvement in nonalcoholic fatty liver disease

**DOI:** 10.1038/s41419-018-0386-3

**Published:** 2018-03-02

**Authors:** Guilherme S. Mazzini, Jad Khoraki, Matthew G. Browning, Guilherme M. Campos

**Affiliations:** 1 Department of Gastrointestinal Surgery, Hospital de Clinicas de Porto Alegre, 2350 Ramiro Bracelos Street, Porto Alegre, RS Brazil; 2 Department of Surgery, Virginia Commonwealth University, 1200 East Broad Street, Richmond, VA USA

Nonalcoholic fatty liver disease (NAFLD) is strongly associated with obesity, metabolic syndrome, and type 2 diabetes (T2DM), which is projected to become the leading cause of liver-related morbidity and mortality within 20 years^1^. Nuclear receptor dysregulation contributes to the pathogenesis of NAFLD by impacting the energy and nutrient-integrated control of metabolism and inflammation, and ligand-activated nuclear receptors have been studied as targets for novel NAFLD therapies^2^. Thus, we read with great interest the paper previously published in *Cell Death & Disease*, entitled “miR-21 Ablation and Obeticholic Acid Ameliorate Nonalcoholic Steatohepatitis in Mice”, by Rodrigues et al.^3^. Liver miR-21 is one of the most upregulated microRNAs in nonalcoholic steatohepatitis (NASH) patients^4^ and is a potent inhibitor of peroxisome proliferator-activated receptor α (PPARα), a nuclear receptor, which expression in liver is decreased in NASH patients^5, 6^. The authors demonstrated that the wild-type (WT) mice fed a methionine and choline-deficient (MCD) diet developed steatohepatitis, presenting moderate to severe vacuolation of hepatocytes, large lipid droplets, pronounced hepatocellular hypertrophy, and moderate to severe inflammation. These changes were almost abrogated in miR-21 knockout (KO) mice, which exhibited markedly reduced steatosis. miR-21 ablation led to increased liver PPARα, with miR-21 KO mice presenting mild to moderate hepatocellular vacuolation, smaller and scattered lipid droplets, as well as a robust decrease in cell hypertrophy.

However, Loyer et al.^5^ previously demonstrated that either pharmacologic-inhibiting or knocking-out miR-21 reduced liver cell injury, inflammation and fibrosis in mice fed an MCD diet, but had no effect in steatosis. Liver lipid accumulation and liver expression of ß-oxidation-related genes were not different between miR-21 KO and WT mice. They also studied liver miR-21 cell localization in liver. In both mice and human patients with NASH, miR-21 was primarily overexpressed in biliary (CK19 + ) and inflammatory (CD3 + ) cells, but not in hepatocytes. We encourage Rodrigues et al. to address this main difference between their results and the previous one from Loyer et al. regarding lipid accumulation, as they used a similar experimental model. Taking into account that Loyer et al. detected the overexpression of miR-21 mainly in biliary and inflammatory cells in liver, it is reasonable to expect the improvement in inflammation and fibrosis, but not in steatosis. Lipid accumulation is a consequence of hepatocyte metabolism impairment, and only the modulation of PPARα inside hepatocytes could lead to the improvement in steatosis. Diet-induced obese mice treated with a PPARα agonist improved hepatic steatosis accompanied by enhancement of the hepatocyte ultrastructure favoring β-oxidation and decrease in hepatic de novo lipogenesis^7^, but no study has compared miR-21 KO to PPARα agonist treatment. Francque et al.^8^ first demonstrated that human liver PPARα gene expression negatively correlates with NASH severity and insulin resistance, and suggested PPARα as a potential therapeutic target in NASH. However, in our opinion, the role of miR21 inhibition in hepatocyte PPARα activation should be better investigated.

Finally, in the same study, Rodrigues et al. also demonstrated that the simultaneous miR-21 suppression and Farnesoid X receptor (FXR) activation by obeticholic acid had a greater impact in preventing NASH development than both treatments separately, in mice fed a fast-food diet^3^. Nuclear receptor-targeted therapies have shown to be beneficial for NAFLD experimentally, but the clinical effectiveness is still unsatisfactory. A novel pharmacological strategy by multi-target nuclear receptor modulation may be the optimal way to obtain benefit from nuclear receptor activation while minimizing adverse effects.
